# Reversed-Image Mechanical Thrombectomy for Acute Ischemic Stroke in a Situs Inversus Totalis Patient

**DOI:** 10.7759/cureus.55629

**Published:** 2024-03-06

**Authors:** Keita Tamada, Hiroki Sato, Satoshi Iihoshi, Yukihiro Imaoka, Shinichiro Yoshikawa, Hiroki Kurita, Shinya Kohyama

**Affiliations:** 1 Department of Endovascular Neurosurgery, Saitama Medical University International Medical Center, Saitama, JPN; 2 Department of Neurosurgery, Saitama Medical University International Medical Center, Saitama, JPN

**Keywords:** situs inversus totalis, image inversion technique, acute ischemic stroke, left-right reversal system, mechanical thrombectomy

## Abstract

An 85-year-old female with situs inversus totalis presented with right hemiplegia, right facial nerve palsy, eye deviation to the left, and aphasia. Magnetic resonance imaging revealed acute ischemic lesions in the left insular cortex and the frontal lobe. Magnetic resonance angiography revealed an occlusion of the left internal carotid artery. Reversed-image mechanical thrombectomy achieved complete reperfusion in three passes within 54 minutes. Six months post-intervention, the patient could walk indoors independently. Our technique, which replicates the normal arterial anatomy by inversion and angulation, was adapted to situs inversus totalis.

## Introduction

Situs inversus totalis is one of the human laterality disorders characterized by the abnormal location and orientation of thoracoabdominal organs and vessels across the left-right axis. The occurrence of situs inversus totalis is in one out of 6,500-25,000 patients [1]. In general, situs inversus totalis is asymptomatic, and many cases are healthy and unaware of it except when comorbid with primary ciliary dyskinesia. When performing mechanical thrombectomy for acute ischemic stroke with situs inversus totalis, it is necessary to consider the special vascular structure of the access route and associated technical difficulties. To resolve the technical difficulties, we described a successful mechanical thrombectomy for acute ischemic stroke with situs inversus totalis using an image inversion technique.

## Case presentation

**Figure 1:**
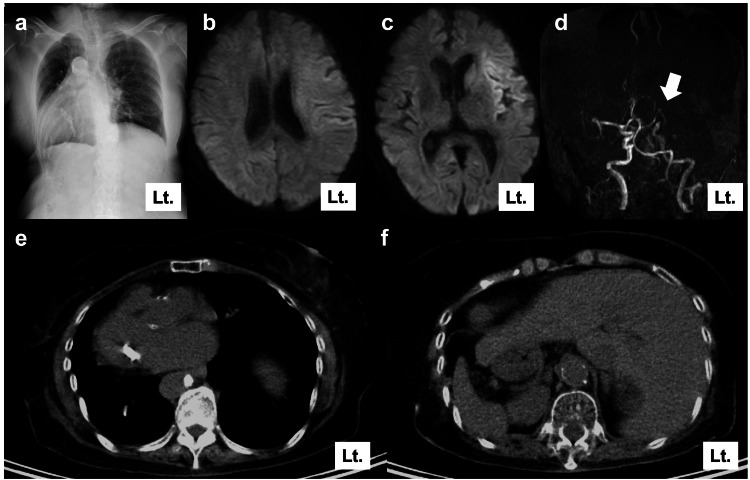
Pre-procedural findings a: Chest X-ray showing a dextrocardia. b, c: Magnetic resonance imaging showing a hyperintense area in the left insular cortex and left frontal operculum. d: Magnetic resonance angiography imaging showing left distal internal carotid artery occlusion (white arrow). e, f: Chest computed tomography showing complete situs inversus totalis obtained during breast cancer treatment

An 85-year-old female with right hemiplegia, right facial nerve palsy, conjugate eye deviation to the left, and aphasia was transported to our hospital as an emergency. The time from the last known well to the emergency department was 90 minutes. She had a history of left breast cancer and a hypertensive cerebellar hemorrhage. The pre-admission modified Rankin scale score was 0. The chest X-ray showed a situs inversus totalis (Figure 1a). Magnetic resonance imaging revealed an ischemic core in the left insular cortex and frontal operculum. Thus, the diffusion-weighted imaging Alberta stroke program early computed tomography score was 8 (Figure 1b-1c). Magnetic resonance angiography revealed occlusion of the left internal carotid artery (Figure 1d). Chest computed tomography performed at the time of breast cancer treatment indicated situs inversus totalis (Figure 1e-1f).

**Figure 2:**
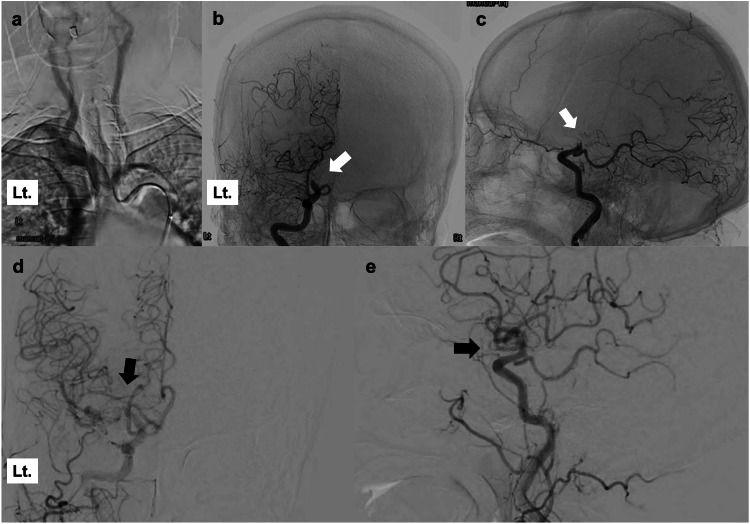
Intra- and post-procedural findings a: Reversal imaging of aortic arch angiography showing an innominate artery originating from the left side. b, c: Reversal imaging of cerebral angiography showing occlusion of the distal internal carotid artery (white arrow). d, e: Reversal imaging of cerebral angiography showing complete recanalization of the left internal carotid artery after mechanical thrombectomy (black arrow)

Mechanical thrombectomy was performed without alteplase because of the pre-existing cerebellar hemorrhage. The patient had situs inversus totalis; therefore, all procedures were performed with reversal images using an Allura Xper FD20/10 (Philips Healthcare, Amsterdam, Netherlands) after the puncture (Figure 2a-2c). The right femoral approach was performed using a 9Fr OPTIMO (TOKAI Medical Products, Kasugai, Aichi, Japan) and a 5Fr JB2 125 cm (Medikit, Tokyo, Japan) in a coaxial position. OPTIMO was advanced to the cervical portion of the left (right on the reverse image) internal carotid artery, and internal carotid angiography revealed occlusion in the C1 portion. Lesion cross was conducted using Phenom 21 (Medtronic, Tokyo, Japan) and Synchro 14 soft (Stryker Neurovascular, Fremont, California, USA), and complete recanalization of the thrombolysis in cerebral infarction grade 3 was achieved after the third pass with a combined technique using Embotrap III 6.5*45 (CERENOVUS, Johnson & Johnson Medical Devices, Irvine, California, USA) and Penumbra ACE68 (Penumbra Inc., Alameda, California, USA). The time from the puncture to recanalization was 54 minutes (Figure 2d-2e). The patient's aphasia improved, and her right paralysis recovered. She was transferred to a rehabilitation hospital with a modified Rankin scale score of 4 after 42 days after the onset. Six months later, the patient recovered to the level of being able to walk indoors without assistance.

## Discussion

In this case, during mechanical thrombectomy in a patient with situs inversus totalis, all fluoroscopic images were reversed from right to left after femoral artery puncture. This allows normal technical manipulation and reduces the discomfort experienced by surgeons.

The vascular anatomy of situs inversus totalis is often a mirror image configuration, and most surgeons are unfamiliar with its access routes and catheter manipulation. In the field of cerebrovascular disease, catheterization of situs inversus totalis is rarely reported, and there are no reports on special techniques from the aortic arch to the upper part of the aortic arch. However, some techniques have been reported in the cardiovascular field, including percutaneous coronary intervention [2,3]. To avoid misinterpretation due to variations in the vascular anatomy of situs inversus totalis, Goel et al. [3] reported that inversion of the left and right sides of the image and modification of the angle, known as the double inversion technique, can be performed in the same manner as in the normal heart. Following their report, we performed only a left-right inversion after the puncture, as if it were a normal surgery. Minor angle modifications may be of little importance in mechanical thrombectomy for acute ischemic stroke. In patients with cerebral arteriovenous malformations, we performed intraoperative cerebral angiography using a reversed image in the supine position. This allows normal technical manipulation and reduces operator discomfort without complications, similar to mechanical thrombectomy.

In this case, we continued the procedure with the left-right inverted. Above the aortic arch, it may be confusing if subsequent operations are performed on inverted images. However, repeating left-right inversion frequently may also confuse, especially when the guiding catheter falls into the aorta. In mechanical thrombectomy for acute ischemic stroke, it is less confusing because the branch of choice is the relatively thick M2 branch and not the thin branch with its complex configuration. In addition, performing with biplane images may have contributed to less confusion in this case. Therefore, flipping above the aortic arch is also acceptable, but it may not make much technical difference between reversal and normal images in time-restricted mechanical thrombectomy.

There are only two cases of mechanical thrombectomy for acute ischemic stroke with situs inversus totalis. have been reported in the literature [4,5]. They reported that complex aortic arch configurations, such as the right-sided aorta, may make it difficult to advance the catheters into the target vessels. However, they did not mention using any special techniques, from the aortic arch to the cerebral vessels. In terms of carotid artery stenting for patients with a right-sided aortic arch, Ohtani et al. [6] reported that a transbrachial approach should be considered when standard femoral artery access is difficult due to unfamiliar anatomical images of the aortic arch. In contrast to carotid artery stenting, mechanical thrombectomy is time-limited, and changing the puncture site can be problematic. Using our reverse imaging technique may reduce the time required and change the puncture site because of situs inversus totalis.

## Conclusions

All fluoroscopic radiographs were reversed to the right and left sides for the patient with situs inversus totalis, which enabled a normal operation and reduced the surgeon’s discomfort. Because most angiography systems are equipped with a left-right reversal system and do not require any special manipulation by a radiologist, this technique may become a standard procedure for mechanical thrombectomy in patients with situs inversus totalis.
